# Dutch individuals’ views of screening for oesophageal cancer: a focus group study

**DOI:** 10.1136/bmjgast-2023-001136

**Published:** 2023-05-31

**Authors:** Jasmijn Sijben, Yonne Peters, Sharell Bas, Peter Siersema, Linda Rainey, Mireille Broeders

**Affiliations:** 1 Department of Gastroenterology and Hepatology, Radboudumc, Nijmegen, Gelderland, The Netherlands; 2 Department for Health Evidence, Radboudumc, Nijmegen, Gelderland, The Netherlands; 3 Department of Gastroenterology and Hepatology, Erasmus MC, Rotterdam, Zuid-Holland, The Netherlands; 4 Dutch Expert Centre for Screening, Nijmegen, The Netherlands

**Keywords:** Barrett's oesophagus, Barrett's metaplasia, oesophageal cancer, gastroesophageal reflux disease, cancer prevention

## Abstract

**Objective:**

Screening for early oesophageal adenocarcinoma (OAC), including its precursor Barrett’s oesophagus (BO), can potentially reduce OAC-related morbidity and mortality. This study explores Dutch at-risk individuals’ views of screening an at-risk population for BO/OAC.

**Design:**

We invited 372 individuals with risk factors for OAC from primary care practices, 73 individuals with surveillance experience, and 221 participants of previous studies (BO/OAC screening trial or survey) to participate in focus groups. Transcripts were inductively and thematically analysed by two independent researchers.

**Results:**

A total of 50 individuals (42% with gastro-oesophageal reflux symptoms) of 50–75 years participated. Themes that were raised included: theme 1 ‘screening intentions’ describing participants’ motivation to be screened (eg, early diagnosis, potential reassurance, physician recommendation, and knowing someone with cancer) or decline screening (eg, anticipated discomfort or suboptimal accuracy of the test); theme 2 ‘risk-based eligibility’ describing the tension between effectiveness (eg, targeting high-risk individuals) and inclusivity (eg, making screening available for everyone); theme 3 ‘distributive justice’, in which the pressure of a potential new screening programme on healthcare resources was discussed; and theme 4 ‘information needs’ describing the perceived lack of information access and individuals’ preference to discuss screening with their general practitioner.

**Conclusion:**

Individuals not only expressed high willingness to be screened but also voiced the concern that a new screening programme may pressure limited healthcare resources. If implemented, it is crucial to develop educational materials that meet the public’s information needs and explain the test procedures and eligibility criteria while avoiding stigmatising language.

**WHAT IS ALREADY KNOWN ON THIS TOPIC**

- Screening for oesophageal adenocarcinoma (OAC) will only be beneficial if the screening policy is in line with the preferences of the target screening population.
- The public has previously been involved in qualitative and mixed methods studies investigating their acceptance of undergoing transnasal endoscopy, an ingestible cell collection device or breath analysis for this purpose, which has indicated that individuals prefer a screening test that is less discomforting than conventional upper endoscopy and very accurate.
- Insight into at-risk individuals’ views on Barrett’s oesophagus/OAC screening, irrespective of the screening test used, is needed to further guide the design of a screening policy.

**WHAT THIS STUDY ADDS**

- The intention to participate in OAC screening is motivated by belief in its benefits, seeking reassurance, a physician’s recommendation or knowing someone with cancer.
- Sex-specific and race-specific screening guidelines may cause fear of exclusion. Introduction of overweight and smoking as eligibility criteria may be perceived as stigmatising.
- Individuals are worried about the pressure of a new screening policy on healthcare resources.
- Individuals prefer to discuss a risk-based screening offer with a healthcare professional.
- Some individuals may decline to undergo transnasal endoscopy or swallow an ingestible cell collection device for screening due to anticipated discomfort.

**HOW THIS STUDY MIGHT AFFECT RESEARCH, PRACTICE OR POLICY**

- The findings can be used to align screening test options, the risk stratification approach, and the development of information materials with individuals’ preferences, worries, and questions.

## Introduction

Oesophageal adenocarcinoma (OAC) causes approximately 85 000 deaths worldwide annually.^1^ While early OAC (stage I) can be endoscopically resected with excellent outcomes,^2^ 83% of cancers have already passed this stage at diagnosis and require invasive oesophagectomy or cannot be cured.^3 4^ Barrett’s oesophagus (BO), which arises in response to gastro-oesophageal reflux disease (GORD), is a potential target for early detection strategies since it is thought to precede OAC.^5 6^ Multiple western gastroenterology guidelines have therefore suggested to screen at-risk individuals (with GORD, age>50 years, male sex, white race, smoking, obesity, and family history of BO or OAC) for the presence of BO, followed by endoscopic surveillance to timely detect dysplasia or early OAC.^7 8^

Studies exploring the public’s perspective on BO/OAC screening mainly addressed their views on screening test alternatives for conventional endoscopy, which is considered too invasive and costly to screen large populations. In the UK, qualitative studies suggested that individuals with GORD preferred the Cytosponge-trefoil factor 3 technology over conventional endoscopy, although participants expressed concerns about swallowing and pulling back the sponge.^9–11^ Discrete choice experiments confirmed that Dutch individuals prefer less-invasive tests under the condition that the sensitivity and specificity are over 80%.^12 13^ Furthermore, interviews revealed that a transnasal (ultrathin) endoscopic approach increased the comfort level for BO patients and made them feel more empowered during the procedure, although they had concerns regarding its thoroughness.^14^

To guide the design of an effective screening strategy, it is important to gain a more comprehensive understanding of individuals’ general willingness to be screened for BO/OAC irrespective of the test used. Previous surveys and screening trials suggested that the presence of GORD symptoms and easily accessible test options may play an important role in screening motivation.^13 15–17^ However, the potential influence of socioeconomic factors, social stigma, and involvement of the general practitioner on willingness to be screened for BO/OAC remain largely untouched.^18^

We aimed to explore Dutch at-risk individuals’ perceptions of screening an at-risk population for BO/OAC. Our secondary aim was to collect their perceptions of novel screening tests, namely: transnasal endoscopy, ingestible cell collection devices, and breath analysis.

## Methods

### Study design and setting

Focus groups (FGs) following a semistructured interview guide were performed to explore individuals’ perceptions of screening for BO/OAC. Due to COVID-19 regulations during the start of the study, FGs were planned online using video conferencing software Zoom. The study and its findings are reported in accordance with the Consolidated Criteria for Reporting Qualitative Studies: 32-item checklist (online supplemental table S1).^19^

10.1136/bmjgast-2023-001136.supp2 Supplementary data

### Participants and recruitment

Since the exact target population for BO/OAC screening is undecided, we defined three categories of interest: (1) members of the general public without GORD that have at least one other risk factor for OAC (male sex, age>50 years, white race, central obesity, smoking history or familial presence of BO/OAC); (2) individuals with GORD, regardless of other risk factors; and (3) individuals with BO (for their surveillance experience). Eligible participants were men and women aged 50–75 years who were fluent in Dutch, belonged to one or more of the categories of interest, and had access to the Zoom platform using a phone, tablet or computer. Eligible individuals were purposively sampled to ensure diversity in characteristics regarding gender, age group, education level, ethnic background, and lifestyle (body mass index, smoking, and alcohol consumption).

Eligible individuals were recruited using the following four different approaches. (1) We selected individuals with and without GORD (and at least one risk factor for OAC) in an even distribution using electronic patient files from primary care practices. Patients with GORD were identified using Integrated Primary Care Information codes for heartburn (D03) and oesophageal reflux disease (D84), and by searching for acid-suppressant prescriptions. The primary care practices were located in Oosterhout (a village in a rural area) and Lent (a neighbourhood in the city of Nijmegen), the Netherlands. Socioeconomic status (SES) scores of Oosterhout and Lent are 0.27 and 0.41, respectively. SES scores are published by Statistics Netherlands (CBS) and based on financial welfare, education attainment, and employment; the score ranges from −0.9 (most deprived) to 0.9 (least deprived), with 0 being the Dutch average.^20^ (2) Individuals with BO were further selected from the surveillance registry of the department of gastroenterology at the Radboudumc, Nijmegen, the Netherlands. This registry includes people from unurbanised to strongly urbanised areas with varying SES scores. (3) We also invited participants from the ELECTRONIC study (a screening trial in which individuals with GORD were invited to undergo breath analysis and transnasal endoscopy to screen for BO)^21^ and a previous survey about familial BO and OAC.^22^ Individuals selected through approaches 1–3 received an electronic invitation letter from their general practitioner or the research team and a reminder after 2 weeks. (4) We further distributed information leaflets in municipal buildings and flats in deprived neighbourhoods in Nijmegen (Lindenholt and Hatert; SES scores of −0.45 and −0.53) to target sections of the community that are difficult to involve in public health participation, such as minority groups, people with health literacy difficulties, and the service resistant.

### Data collection

FGs took place between January and June 2022. We involved three representatives of the Dutch advocacy group for people with literacy difficulties (foundation ABC) in the development of the information letter. The FGs followed a semistructured interview guide, which was based on a systematic literature review.^18^ A complete version of the interview guide is available in online supplemental file 1. The FGs started with exploratory questions about participants’ general impressions and perceived risk of OAC. Participants were subsequently educated about BO, oesophageal cancer, the effect of GORD on BO/OAC development, current surveillance methods, and the option to offer at-risk individuals (based on criteria such as: GORD, male sex, age>50 years, obesity, western background, familial presence, smoking, alcohol consumption) a screening test to identify people with BO. Selection methods (eg, varying eligibility criteria proposed by different guidelines, intensifying screening for white men or application of a risk calculator) were not within the scope of this study. Participants were then encouraged to share their thoughts regarding BO/OAC screening and how they felt this would impact their lives. Lastly, we elicited participants’ views of various screening tests, after educating them about the purpose, location, procedures, accuracy, and potential consequences of each test. Test accuracy was framed as ‘very good’, ‘good’, ‘reasonable’, and ‘moderate’ for conventional upper endoscopy, transnasal endoscopy, ingestible cell collection devices, and breath analysis, respectively. FGs were organised until data saturation was achieved, that is, no new themes were identified. Duration of the FGs ranged from 64 to 112 min. Group size ranged from 4 to 10 participants. The groups were both homogenous and heterogenous in relation to participants’ previous experiences with BE and screening tests (online supplemental table S2). Each FG was video recorded and transcribed verbatim. Participants also completed a short questionnaire on demographics and health background.

10.1136/bmjgast-2023-001136.supp1 Supplementary data

All FGs were moderated by JS and the first FGs were supervised by LR. JS is a female medical doctor and has been trained in qualitative interviewing. LR is a female psychologist and epidemiologist with extensive experience in qualitative interviewing. One moderator (JS) had met two participants prior to the study. Participants were given no information about the moderators beyond their educational background and research interests.

### Data analysis

We inductively analysed the data using Braun and Clarke’s method of thematic analysis, that is, familiarisation with the data, coding, developing, reviewing, defining, and naming themes, and final analysis.^23^ Two researchers (JS and SB) independently analysed all transcripts in ATLAS.ti (V.7.1.5). When discrepancies arose, consensus was reached through discussion with the entire research team.

### Public involvement

This study was conducted to involve the public in the design of an BO/OAC screening strategy. Participants did not contribute to the interpretation or writing of the findings but were provided (if they desired) a lay summary of the final results. The results will also be presented during a patient symposium.

## Results

### Summary

Of the 666 individuals invited to take part, 50 participated (7.5%). Participation rates per recruitment approach were: 25 of 372 invited through primary care practices (6.7%); 17 of 73 BO surveillance patients (23.3%); 8 of 221 individuals (3.6%) participating in studies related to screening for BO/OAC (screening trial or survey). Distribution of leaflets did not result in additional participants. A total of 8 FGs were conducted (50 participants), with group sizes ranging from 4 to 10 participants. Table 1 shows background characteristics of the participants. Forty-two percent had GORD symptoms and around a third (36%) knew someone who had oesophageal cancer.

**Table 1:** General characteristics of the study population

Characteristic	All participants (N=50)	Primary care participants (N=25)	BO registry participants (N=17)	Previous study participants (N=8)
Age (years), mean (range)	63.7 (50–75)	60.8 (50–75)	67.0 (56–75)	66.0 (60–75)
Male gender, n (%)	29 (58.0)	15 (60.0)	10 (58.8)	4 (50.0)
White ethnicity, n (%)	48 (96.0)	24 (96.0)	16 (94.1)	8 (100.0)
Highest level of education, n (%)				
Primary/high school	11 (22.0)	5 (20.0)	2 (11.8)	4 (50.0)
Vocational college	11 (22.0)	7 (28.0)	4 (23.5)	0 (0.0)
College or university	28 (56.0)	13 (52.0)	11 (64.7)	4 (50.0)
Current employment, n (%) yes				
Working full-time	14 (28.0)	12 (48.0)	1 (5.9)	1 (12.5)
Working part-time	10 (20.0)	3 (12.0)	4 (23.5)	3 (37.5)
Unemployed, looking for work	1 (2.0)	1 (4.0)	0 (0.0)	0 (0.0)
Retired	25 (50.0)	9 (36.0)	12 (70.6)	4 (50.0)
Current GORD symptoms,* n (%) yes	21 (42.0)	11 (44.0)	7 (41.2)	3 (37.5)
Upper endoscopy experience, n (%) yes	45 (90.0)	21 (84.0)	17 (100.0)	7 (87.5)
PPI usage, n (%) yes	42 (84.0)	21 (84.0)	16 (94.1)	5 (62.5)
BO diagnosis, n (%) yes	17 (34.0)	0 (0.0)	17 (100.0)	0 (0.0)
Knowing someone with oesophageal cancer, n (%) yes	18 (36.0)	9 (36.0)	5 (29.4)	4 (50.0)
Participant ELECTRONIC study (screening trial), n (%) yes	8 (16.0)	1 (4.0)	4 (23.5)	3 (37.5)
Participated in population-based cancer screening programmes, n (%) yes	39 (78.0)	18 (72.0)	14 (82.4)	7 (87.5)

*Based on a GerdQ^32^ Score of 8 or higher.

GORD, gastro-oesophageal reflux; PPI, proton pump inhibitor; BO, Barrett’s oesophagus.

**Figure 1:**
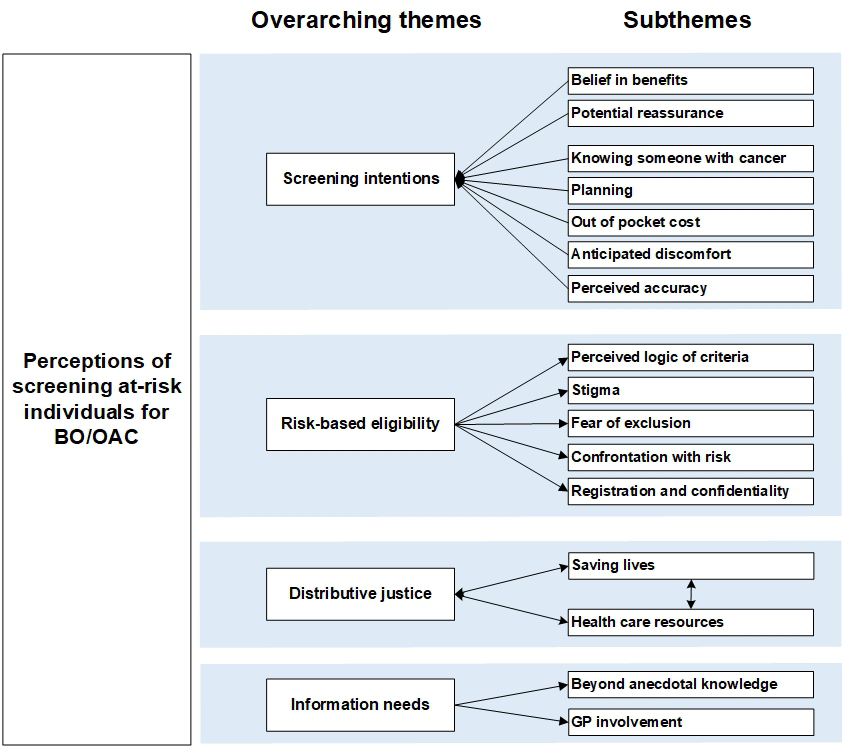
Themes and subthemes. BO, Barrett’s oesophagus; GP, general practitioner; OAC, oesophageal adenocarcinoma.

We derived four themes from the thematic analysis, that is, screening intentions, perceived logic of eligibility, distributive justice, and information needs (figure 1). Direct quotes from participants (translation in online supplemental table S3) illustrate the themes and subthemes in the following section. Subthemes are listed in italics in the text. Perceptions specific to each of the discussed screening tests are summarised in table 2.

**Table 2:** Summary of codes describing individuals’ perceptions of transnasal endoscopy, ingestible cell collection devices, and breath analysis

	Codes	Exemplary quotes
Transnasal endoscopy	From some to high (anticipated) discomfort	They can numb the nose, so that the tingle in the nose is gone. (FG5, female, 69 years, no BO) I personally had a test to examine my vocal cords, which also goes through your nose and reaches quite far back. I actually didn't find that too burdensome. (FG3, female, 53 years, no BO) I mean, it won’t make the gagging any better or worse, right? (FG2, female, 53 years, no BO) Honestly, well, I’ll get through it with gritted teeth, but I dread it more than via the mouth. (FG7, male, 74 years, BO)
	Organisational implications	Yes, I think you would need more staff with a test via the mouth than via the nose. I think the one via the nose can be done by one person. (FG7, female, 68 years, BO) The person who examines it must be able to judge it very well. (FG1, female, 69 years, BO) This way you pre-select, because you can’t perform population-based screening in a hospital, that is simply not possible. But maybe it’s doable with a mobile unit. And if nothing is detected, you don’t have to go to hospital. So I think it decreases the burden on health care. (FG2, male, 75 years, no BO)
	No surplus for the public	What’s the improvement? That it is five millimetres? It still goes down your throat. It first goes through your nose and instead of undergoing it once you may have to do it twice. The difference is in the sedation. I wonder, is that really a considerable improvement? (FG1, female, 59 years, BO) ‘A simple test’, I mean something else by that. (FG6, male, 50 years, no BO)
Ingestible cell collection device	From no to high anticipated discomfort	Just imagine it’s a delicious piece of candy, and it’ll go right down. All done. Right? (FG4, male, 69 years, no BO) I assume that it is the same as taking your pills. (FG2, male, 55 years, no BO) I’m not convinced. I think that the piece of string in your throat, which is something that shouldn’t be there, will automatically cause a lot of irritation and gagging. It’s like when the dentist uses a mirror to check your molars and he gets close… (FG2, male, 75 years, no BO) Unpleasant. (FG5, male, 72 years, no BO) People will pull that thing out in no time. So from my experience, I'm really like: this isn't going to work. (FG5, female, 69 years, no BO)
	Worries about the procedure	But if that has to go up through your throat, that will close things off for a while. (FG3, female, 53 years, no BO) I’m just thinking: how can you swallow a 2 cm long, how wide is it, a centimeter in circumference? How could you get that down your throat? (FG5, male, 60 years, no BO) It also has an adhesive effect. To what extent does your voice, what are they called, do the vibrating fibres of your voice stick to it? Your vocal cords are made up of very fine fibres. To what extent do they stick to the sponge when you retrieve it and thus possibly damage your voice? (FG2, male, 75 years, no BO) But that piece of string, I don't know, if I swallow, how far does that medicine get into the oesophagus on the first swallow? Will it drop down by itself, also past my valve? (FG7, male, 70 years, no BO) That pill can get stuck halfway, and you’ll be like: I can't get it any further. (FG5, female, 63 years, BO) Well, if the piece of string snaps, I don't know where I'd rather be. (FG3, female, 50 years, no BO)
	Thoroughness	Yeah, I think, that cancer, Yeah, I still think that the cancer starts in a certain place. And that sponge, yeah, it scrapes cells along with it, but it may miss the cancerous cells. So that would be a missed opportunity. (FG7, male, 74 years, BO)
Breath analysis	Least burdensome	If I get to choose, let me see, what would I find the most comfortable? That would of course be the breath test, that’s true. But, you know, I find accuracy more important. (FG3, female, 57 years, BO) There is less risk of damage. The other tests can all cause damage, taking a biopsy, if something is just a little off. (FG2, female, 67 years, no BO)
	Convenience	In principle it is a good method, because it is probably quite affordable, you should also consider that. (FG7, male, 74 years, BO) Because it is easily accessible, you can even do it at the GP’s office. (FG4, male, 53 years, no BO)
	Accuracy is a prerequisite	I'm not thrilled by such a test, let’s put it that way. Look, if the GP says: 'just breathe into this device ’, then you’ll do that. But I won't be reassured after I hear: 'there’s nothing wrong'. So that’s a real issue. In the end, you want to tell people, well, 'certainty' is a big word, but you still want to relay results in a reliable way. I'm not eager to participate with this test. (FG4, male, 52 years, no BO) If it isn’t reliable, I’m sorry to say, but then it’s completely useless. (FG2, female, 67 years, no BO)
	Understanding	Is this for oesophageal cancer or is it also for that… now I've forgotten the term again, that Barrett? (FG4, male, 53 years, no BO) I was curious actually: what does it measure? The stomach acid or something? The acidity of the stomach? Blood spatters? (FG8, male, 65 years, no BO) Okay, so you could measure whether you have oesophageal cancer via your lungs… Even though that is another system, right? (FG5, female, 62 years, no BO)

BO, Barrett’s oesophagus; FG, focus group; GP, general practitioner.

### Theme 1: screening intentions

Both intrinsic (*belief in benefits, potential reassurance*) and extrinsic factors (*knowing someone with cancer, planning, out-of-pocket cost, anticipated discomfort and perceived accuracy of the screening test*) influenced the intention to participate in BO/OAC screening.

Participants expressed a profound belief that having your oesophagus checked through screening could help to prevent cancer or detect it more quickly (*belief in benefits*). Some participants cited participation in cancer screening as a habit.

Yes, I am in favour of any available test and population-based screening program, with which you can intervene properly through early detection. (FG7, female, 68 years, BO)

Many participants, especially those with frequent GORD symptoms, worried about gastric acid damage and felt screening could give them *reassurance*. For these participants, ‘peace of mind’ was a key benefit motivating screening uptake.

Suppose you have symptoms, if there is a simple test that can take away your anxiety, that would be nice. (FG6, female, 62 years, no BO)

Another motivation people gave for intending to participate was *having a close family member or friend who had cancer*. Discouraging factors included being too busy and forgetting to schedule the test (*planning*). One participant mentioned that a phone call reminder would help. Willingness to pay *out-of-pocket cost* was mixed and depended on income and the price of the test.

Intention to participate in screening was also entangled with *anticipated discomfort* and *perceived accuracy* of each screening test (also see table 2).

Participants expressed varying levels of anticipated physical discomfort regarding transnasal endoscopy. Although participants recognised some organisational advantages, the majority were unsure if a nasal approach would be an actual improvement since they expected the discomfort to be similar to conventional upper endoscopy. According to participants, the test has no surplus if it must be confirmed by upper endoscopy.

The introduction of ingestible cell collection devices elicited worries regarding discomfort and accuracy, such as: fear of gagging, inability to swallow the device, breaking of the string or the device missing cancer cells.

Breath analysis was viewed as a convenient and painless test. However, suboptimal accuracy was a concern. Participants unanimously stated that high test accuracy is essential for screening intent. They felt that a test with a high risk of a false-negative result is useless, since it may lead to false reassurance and risk of cancer progression. A few participants mentioned the psychological impact of a false-positive test result.

### Theme 2: risk-based eligibility

Most participants would like screening to become available to everyone. Some thought screening a smaller group at a higher risk of developing oesophageal cancer may be more realistic. The acceptability of at-risk screening relied on the *perceived logic* of the association between a risk factor and oesophageal cancer. Participants expressed particularly high understanding of why gastric reflux predisposes to OAC.

[Before education] I immediately link that to the issues heartburn causes. Eventually, gastric acid can also lead to wounds in your oesophagus. And I believe that is the cause of cancer. (FG4, male, 52 years, no BO)

In contrast, some participants denied the link between obesity, smoking or drinking alcohol and oesophageal cancer or health problems in general.

I don't smoke and I also have it [Barrett’s oesophagus], so how so? You know, that just doesn't make sense to me. (FG1, female, 59 years, BO)

I was healthier when I weighed 66 pounds more. (FG2, female, 53 years, no BO)

Participants were also concerned that basing screening eligibility on obesity, smoking or drinking may be perceived as *stigmatising,* which could lead individuals to decline the invitation.

Well, if you say: ‘you are in a risk group because you drink alcohol or smoke’, for example, then you are instantly stigmatizing, in my opinion. (FG2, male, 75 years, no BO)

I wouldn't take the risk of some people saying: 'Yeah, piss off, now I'm suddenly too fat… I'm not fat at all, I'm happy.' People won’t get tested. (FG4, male, 52 years, no BO)

The risk factors ‘male gender’ and ‘white ethnicity’ elicited resistant reactions due to fears that women and non-white individuals would be *excluded* from screening.

But would you really exclude women? I think that would be very bad. (FG5, female, 69 years, no BO)

[About the list of risk factors] I would prefer it if Eastern cultures are also mentioned. (FG7, male, 70 years, no BO)

Participants further mentioned that receiving information about their personal risk of developing oesophageal cancer could be *confrontational* and may lead to additional worry compared with generalised screening.

And that is a big difference with mammography and bowel cancer screening, which are age-related. Yeah, then we just know: I will get a call for it at some point. But in this case, you’ll think: oh, so apparently I belong to a certain group that is at higher risk. (FG8, female, 60 years, no BO)

Inadequate *registration* of reflux symptoms, weight, and smoking and drinking behaviour in medical files was an additional concern. *Confidential* treatment of risk information is crucial to individuals; some participants were worried that risk information might end up with insurance companies.

### Theme 3: distributive justice

Although most participants personally intended to participate in BO/OAC screening, concerns regarding the societal consequences of a new screening programme were also evident. Participants particularly valued fair and appropriate distribution of healthcare resources within the society. However, expectations about how BO/OAC screening would stimulate distributive justice were mixed. Some participants valued *saving a human life* over additional costs when considering this issue.

And then I think to myself: what’s the value of money, guys? I think a human life is worth more, that’s worth spending some money on. (FG2, male, 55 years, no BO)

One participant added that good screening options should be available for any type of cancer.

And everyone to whom it happens, would like proper testing for their type of cancer, and have it be curable. (FG8, female, 60 years, no BO)

Some participants, however, concluded that screening for every type of cancer may not be feasible. They worried that the introduction of a new screening programme would *overload healthcare facilities* and personnel.

The GP will probably be flooded again with people who think they are eligible for this. (FG2, female, 53 years, no BO)

Participants were also divided on the economic consequences of introducing BO/OAC screening. Some believed that screening would actually save money for society, due to reduced costs of surgery, chemotherapy and radiotherapy, hospital admission, and sick leave. Others believed the cost of screening would not outweigh these savings. Their support for the allocation of resources for BO/OAC screening, sometimes referred to as their tax money, was finite and depended on how expensive screening would be, the frequency of diagnosis and the number of lives saved.

And to pay a lot of money to maybe save one person’s life, I don't know if we should do that. Then health care in the Netherlands would really become unaffordable. (FG5, male, 72 years, no BO)

### Theme 4: information needs

There were varying levels of knowledge about OAC. Most participants were familiar with alarm symptoms such as difficulty swallowing and food impaction. Other perceived symptoms included hoarseness, earache, and pain between the shoulder blades. Perceived risk factors included heartburn, stress, eating spicy food, genetic/familial presence, drinking alcohol, smoking, drinking hot beverages, and air pollution. Some participants mentioned they were unaware of symptoms or risk factors. Participants who were familiar with alarm symptoms, risk factors, and the severity of oesophageal cancer mainly relied on *anecdotal knowledge*.

My mother-in-law, she had a friend and her husband had oesophageal cancer, and she was seriously upset about it, because the chances of survival are not that great. (FG8, female, 60 years, no BO)

Participants across groups expressed that there is a lack of awareness about oesophageal cancer, risk factors, symptoms, and prognosis in their community. Some participants suggested launching educational campaigns using mass media platforms and warnings for the risks of GORD on antisuppressant medication labels.

I have another point. Shouldn't there first be a large-scale educational campaign in the Netherlands about oesophageal cancer? Most people are not aware of it, don't know it. (FG8, male, 68 years, no BO)

However, others emphasised that informing the public about oesophageal cancer may also increase cancer worry.

Most of our participants would prefer to learn about and discuss BO/OAC screening with their general practitioner (GP). The value of *GP involvement* is centred on trust, familiarity, convenience, and a personal approach.

See, if it were general population-based screening, I wouldn’t have to discuss anything. But if only a select group is screened, then I would like to know why I am part of that group. And then I would really prefer hearing it from my GP. (FG6, male, 50 years, no BO)

## Discussion

The present study provides an overview of Dutch individuals’ views of adopting BO/OAC screening. It showed that, overall, individuals express a high willingness to be screened for oesophageal cancer, although the acceptability of targeted screening strategies and using transnasal endoscopy, ingestible cell collection devices, and breath analyses for screening was mixed.

The main motivators for attending BO/OAC screening (belief in benefits, potential reassurance, GP recommendation, and knowing someone with cancer) have a general character. These factors were previously described in models of screening behaviour^24^ and were observed in established cancer screening settings.^25^ General screening barriers identified in this study were related to planning and costs.^24^ However, there were differences with the literature. Although our participants expressed some cancer worry, none described it as a barrier to attending screening. This finding implies that worry operates mainly as a facilitator in BO/OAC screening and supports Young and Robb’s theory that moderate levels of cancer worry are more motivating than low levels (no drive) or high levels (avoidance).^25^

BO/OAC-specific screening barriers were mainly related to characteristics of the proposed novel screening tests, which was somewhat unexpected given the reported enthusiasm for these tests in previous studies.^9–11 14^ Participants in former studies generally felt that a nasal endoscopic approach or ingestion of a cell collection device is associated with less discomfort than conventional endoscopy,^9 11 14^ whereas our participants were unsure about the potential for improvement. The discordance may have arisen from the hypothetical nature of our study, for example, anticipated discomfort of ingestible cell collection devices was shown to be higher than experienced discomfort.^11^ Similarly, the few participants in our study who had experienced transnasal endoscopy found it less uncomfortable than expected. Nevertheless, the findings indicate that some invitees might be deterred by anticipated discomfort of ingestible cell collection devices and transnasal endoscopy. Therefore, to optimise uptake of ingestible devices, education about the size of the device, risk of gagging, low risk of string breakage and physical damage, and test duration is essential, for example, by showing a demo video.^9^ Transnasal endoscopy was found to be only acceptable to individuals with high screening motivation under the condition that it is performed without confirmatory conventional endoscopy.

Test accuracy was prioritised by individuals in this and former studies.^12 13^ Therefore, the fact that we described the accuracy of breath analysis as ‘reasonable’ and ‘under investigation’ (according to the current literature)^26^ likely explains that participants had doubts about this test. This is in line with a previous study that showed breath analysis was only acceptable if sensitivity and specificity are well above 80%.^12^ These findings stress that breath analysis could be a very suitable pre-test for individuals, including those deterred by invasive options, but only if the artificial intelligence model that supports it achieves high sensitivity and specificity in validated cohorts.

The present study further offers new insights into individuals’ views of risk-based eligibility criteria for BO/OAC screening. This theme exposes a potential disconnect between the perspectives of future providers and consumers of screening, with a tension between effectiveness (targeting high-risk individuals) and inclusivity (making screening available for everyone). A previous study found that screening white men with GORD more often than black men with GORD and not screening women would be optimal in terms of cost-effectiveness.^27^ Yet, participants in our FGs were dissatisfied with sex-specific and race-specific screening guidelines. In addition, an American survey found that black respondents were in fact more worried about developing OAC and more interested in screening than white respondents.^17^ These findings imply that the introduction of socially sensitive eligibility criteria for screening warrants public education on cancer epidemiology to avoid feelings of discrimination and requests from individuals less likely to benefit from screening.

Our results also suggest that selecting obesity as an eligibility criterion may offend people and decrease screening uptake. This aligns with studies on obesity-related media campaigns, which have shown that messages that are perceived as stigmatising instil less motivation to improve health.^28^ Weight-based terminology determines how individuals will react to healthcare messages, with ‘obese’ and ‘fat’ being perceived as stigmatising, whereas a term such as ‘unhealthy weight’ is more likely to promote healthy behaviour.^29^

In terms of societal issues, we observed mixed attitudes depending on whether BO/OAC screening was discussed at a personal or healthcare level, that is, participants being eager to obtain a screening test for themselves but hesitant about the consequences for healthcare resources. This finding is rooted in the construal level theory, which describes that individuals’ thinking differs depending on the psychological distance.^30^ The further away an event is (discussing a future national screening programme), the easier it is for participants to argue rationally and consider the incidence and mortality of cancer, the cost of screening, and the availability of healthcare personnel. The more concrete an event becomes (being informed on increased risk of OAC), the more important emotional arguments such as ‘I want to live as long as possible’ become. It is important to keep in mind that most Dutch citizens are not at high risk for OAC and may thus be less enthusiastic over the introduction of a new screening programme that they cannot benefit from, especially in times of restricted healthcare resources.

### Strengths and limitations

The FG design of this study sparked conversations regarding the adoption of BO/OAC screening, a topic that most participants were unfamiliar with. The purposive sample of participants with diverse characteristics constitutes rich empirical material and provides new insights. The results of this study are limited to a small, self-selecting sample. Non-response is an inevitable problem for any study that is based on voluntary recruitment. We refrained from phone call reminders or offering financial incentives to guarantee selection of individuals that were intrinsically motivated to share their thoughts. The over-representation of individuals with GORD symptoms, higher education, upper endoscopy experience, or who knew someone with oesophageal cancer suggests that self-selection bias is likely to have occurred. These groups are all associated with having more favourable perceptions of BO/OAC screening,^13 15–17 25^ potentially limiting the generalisability of our findings to the general population. Furthermore, patients with BO were prone to a familiarity preference effect,^31^ with individuals tending to prefer tests (conventional endoscopy in this case) merely because they are familiar with it. The inclusion of patients with BO may therefore have influenced perceptions of the novel screening tests. For the above reasons, we are currently performing a survey study aiming for a larger and more representative sample of the general Dutch population to confirm our findings.

## Conclusions and future perspectives

This qualitative study revealed a high intention to participate in BO/OAC screening among individuals, especially those with GORD. Since willingness to accept an invasive test was heterogeneous, offering a variety of test options may yield the highest public interest. Furthermore, surveys (hypothetical or coupled to screening trials) can help to further evaluate public acceptance of risk stratification approaches, such as intensified screening for white men or the application of a risk calculator. If a targeted approach is implemented, it is crucial to develop easily accessible and understandable information materials that explain the eligibility criteria and avoid stigmatising language.

## Data Availability

The data are not publicly available due to privacy restrictions.
