# Supplementary material for: Dutch individuals’ views of screening for oesophageal cancer: a focus group study
Source: BMJ Open Gastroenterol. 2023 May 31;10(1):e001136. doi: 10.1136/bmjgast-2023-001136 (PMC10254588; doi:10.1136/bmjgast-2023-001136)
Supplement: Supplementary data [file bmjgast-2023-001136supp002.pdf]

Table S1. COREQ (COnsolidated criteria for REporting Qualitative research) Checklist.

**Topic**	**Item No.**	**Guide Questions/Description**	**Reported on Page No.**
**Domain 1: Research team and reflexivity**			
Personal characteristics			
Interviewer/facilitator	1	Which author/s conducted the interview or focus group?	6
Credentials	2	What were the researcher's credentials? E.g. PhD, MD	1
Occupation	3	What was their occupation at the time of the study?	6
Gender	4	Was the researcher male or female?	6
Experience and training	5	What experience or training did the researcher have?	6
Relationship with participants			
Relationship established	6	Was a relationship established prior to study commencement?	6
Participant knowledge of the interviewer	7	What did the participants know about the researcher? e.g. personal goals, reasons for doing the research	6
Interviewer characteristics	8	What characteristics were reported about the inter viewer/facilitator? e.g. Bias, assumptions, reasons and interests in the research topic	6
**Domain 2: Study design**			
Theoretical framework			
Methodological orientation and Theory	9	What methodological orientation was stated to underpin the study? e.g. grounded theory, discourse analysis, ethnography, phenomenology, content analysis	6
Participant selection			
Sampling	10	How were participants selected? e.g. purposive, convenience,	5

		consecutive, snowball	
Method of approach	11	How were participants approached? e.g. face-to-face, telephone, mail, email	5-6
Sample size	12	How many participants were in the study?	Table 1
Non-participation	13	How many people refused to participate or dropped out? Reasons?	Table 1
Setting			
Setting of data collection	14	Where was the data collected? e.g. home, clinic, workplace	5
Presence of non participants	15	Was anyone else present besides the participants and researchers?	6
Description of sample	16	What are the important characteristics of the sample? e.g. demographic data, date	Table 1
Data collection			
Interview guide	17	Were questions, prompts, guides provided by the authors? Was it pilot tested?	6
Repeat interviews	18	Were repeat inter views carried out? If yes, how many?	6
Audio/visual recording	19	Did the research use audio or visual recording to collect the data?	6
Field notes	20	Were field notes made during and/or after the interview or focus group?	
Duration	21	What was the duration of the inter views or focus group?	6
Data saturation	22	Was data saturation discussed?	6
Transcripts returned	23	Were transcripts returned to participants for comment and/or correction?	7
**Domain 3: analysis and findings**			
Data analysis			
Number of data coders	24	How many data coders coded the data?	6

Description of the coding tree	25	Did authors provide a description of the coding tree?	N/A
Derivation of themes	26	Were themes identified in advance or derived from the data?	6
Software	27	What software, if applicable, was used to manage the data?	6
Participant checking	28	Did participants provide feedback on the findings?	7
Reporting			
Quotations presented	29	Were participant quotations presented to illustrate the themes/findings?<br>Was each quotation identified? e.g. participant number	7-11<br>Table 2
Data and findings consistent	30	Was there consistency between the data presented and the findings?	7-11<br>Table 2
Clarity of major themes	31	Were major themes clearly presented in the findings?	7-11<br>Figure 1
Clarity of minor themes	32	Is there a description of diverse cases or discussion of minor themes?	7-11<br>Figure 1

Table S2. Composition of participants in each focus group discussion.

Focus group	Participant	Age	Gender	Education	BE diagnosis	Upper endoscopy experience	Electronic participant*
1 (held on 25 January 2022)	110002	70	Male	College or university	Yes	Yes	
	110003	71	Female	College or university	Yes	Yes	
	110004	73	Male	Vocational college	Yes	Yes	
	110006	69	Female	College or university	Yes	Yes	
	110008	66	Male	Primary or high school	Yes	Yes	
	110009	66	Male	Vocational college	Yes	Yes	Yes
	110010	59	Female	Vocational college	Yes	Yes	
	110062	65	Male	College or university	Yes	Yes	
	110063	62	Male	College or university	Yes	Yes	
	110064	75	Male	College or university	Yes	Yes	Yes
2 (held on 31 January 2022)	110015	75	Male	College or university		Yes	
	110021	75	Male	Primary or high school			
	110022	55	Male	Vocational college		Yes	
	110023	51	Female	Vocational college		Yes	
	110026	53	Female	Vocational college		Yes	
	110029	67	Female	Primary or high school		Yes	
	110030	67	Female	College or university		Yes	
3 (held on 24 February 2022)	110017	67	Male	College or university		Yes	
	110020	50	Female	College or university		Yes	
	110031	62	Male	College or university		Yes	
	110033	53	Female	Vocational college		Yes	
	110034	57	Female	College or university	Yes	Yes	
4 (held on 25 February 2022)	110012	53	Male	College or university		Yes	
	110025	51	Male	College or university		Yes	
	110028	55	Male	Vocational college			

	110038	52	Male	College or university		Yes	
	110040	69	Male	Primary or high school		Yes	
5 (held on 7 March 2022)	110039	72	Male	College or university		Yes	
	110042	69	Female	College or university		Yes	
	110045	75	Female	Primary or high school			
	110047	63	Female	College or university	Yes	Yes	
	110052	62	Female	Primary or high school		Yes	
	110053	68	Female	Primary or high school		Yes	
	110055	60	Male	Primary or high school			
6 (held on 8 March 2022)	110016	50	Male	Vocational college		Yes	
	110018	60	Male	Primary or high school		Yes	
	110032	50	Male	College or university		Yes	
	110035	68	Female	Vocational college		Yes	
	110043	75	Female	College or university	Yes	Yes	
	110044	58	Male	College or university		Yes	Yes
	110050	56	Male	Primary or high school	Yes	Yes	
	110057	62	Female	College or university			
7 (held on 20 April 2022)	110048	74	Male	College or university	Yes	Yes	Yes
	110051	70	Male	Vocational college	Yes	Yes	Yes
	110056	75	Female	Primary or high school		Yes	
	110060	68	Female	College or university	Yes	Yes	
8 (held on 9 June 2022)	110065	60	Female	College or university		Yes	
	110066	70	Male	College or university		Yes	Yes
	110068	65	Male	College or university		Yes	Yes
	110069	68	Male	College or university		Yes	Yes

* Experienced breath analysis and transnasal endoscopy.

Table S3. Original quotes with translation.

**Original quote in Dutch**	**English translation**
Theme 1	
Ja, iedere test en bevolkingsonderzoek dat er is, waarmee je door vroege opsporing goed in kunt grijpen, daar ben ik een voorstander van.	Yes, I am in favor of any available test and population-based screening program, with which you can intervene properly through early detection.
Stel je hebt klachten, als er dan een eenvoudige test is die vast je ongerustheid weg kan nemen, dat is ook wel prettig.	Suppose you have symptoms, if there is a simple test that can take away your anxiety, that would be nice.
Theme 2	
Ik link dat gelijk aan de last die maagzuur veroorzaakt. Uiteindelijk, maagzuur kan ook tot wonden leiden in je slokdarm. En dat is juist datgene wat in mijn beleving tot kanker leidt.	I immediately link that to the issues heartburn causes. Eventually, gastric acid can also lead to wounds in your oesophagus. And I believe that is the cause of cancer.
Ik rook niet en ik heb het [Barrett slokdarm] ook aan me, dus hoezo? Weet je, dat zegt gewoon niks in mijn optiek	I don't smoke and I also have it [Barrett's oesophagus], so how so? You know, that just doesn't make sense to me.
Ik was gezonder toen ik 30 kilo zwaarder woog.	I was healthier when I weighed 66 pounds more.
Nou ja, als je gaat zeggen van: u zit in een risicoklas omdat u bijvoorbeeld alcohol drinkt of rookt, dan zit je gelijk te stigmatiseren vind ik.	Well, if you say: ‘you are in a risk group because you drink alcohol or smoke’, for example, then you are instantly stigmatizing, in my opinion.
Ik zou niet het risico nemen dat sommige mensen zeggen: ‘Ja flikker toch op, nu ben ik ineens te dik… Ik ben helemaal niet dik, ik ben gelukkig.’ Dan komen mensen niet testen.	I wouldn't take the risk of some people saying: 'Yeah, piss off, now I'm suddenly too fat... I'm not fat at all, I'm happy.' People won’t get tested.
Maar sluit je vrouwen dan ook echt uit? Dat vind ik een zeer slechte zaak.	But would you really exclude women? I think that would be very bad.
[Over het rijtje risico factoren] Ik zou het fijn vinden als er ook oosterse cultuur bij genoemd wordt.	[About the list of risk factors] I would prefer it if Eastern cultures are also mentioned.
En dat is een groot verschil met mammografie en darmkanker onderzoek, dat gaat leeftijd gerelateerd. Ja, dan weten we gewoon van: ik krijg er op een gegeven moment een oproep voor. Maar dit geval denk je van: oh, ik hoor dus blijkbaar bij een bepaalde groep die groter risico loopt.	And that is a big difference with mammography and bowel cancer screening, which are age-related. Yeah, then we just know: I will get a call for it at some point. But in this case you’ll think: oh, so apparently I belong to a certain group that is at greater risk.
Theme 3	
En dan denk ik bij mijn eigen: ja, jongens, wat is dan geld? Dan vind ik een	And then I think to myself: what’s the value of money, guys? I think a human life is

mensenleven meer waard, dat mag wel wat geld kosten.	worth more, that's worth spending some money on.
En iedereen die het overkomt, wil graag dat er voor zijn vorm van kanker of wat dan ook een goed onderzoek is en dat het genezend is.	And everyone to whom it happens, would like proper testing for their type of cancer, and have it be curable.
De huisarts zal dan ook weer overspoeld worden waarschijnlijk met mensen die denken daarvoor in aanmerking te komen.	The GP will probably be flooded again with people who think they are eligible for this.
En om heel veel te betalen om misschien een persoon te redden, ik weet niet of we dat moeten doen. Dan wordt de gezondheidszorg in Nederland echt onbetaalbaar.	And to pay a lot of money to maybe save one person's life, I don't know if we should do that. Then health care in the Netherlands would really becomes unaffordable.
Theme 4	
Via mijn schoonmoeder, die had weer een vriendin en haar man had slokdarmkanker en daar was zij dus ernstig van ontdaan, want de overlevingskansen zijn niet zo groot.	My mother-in-law, she had a friend and her husband had oesophageal cancer, and she was seriously upset about it, because the chances of survival are not that great.
Ik heb nog een ander punt. Moet er niet eerst gewoon überhaupt een uitgebreide voorlichtingscampagne komen in Nederland ten aanzien van slokdarmkanker. De meeste mensen kennen het niet, weten het niet.	I have another point. Shouldn't there first be a large-scale educational campaign in the Netherlands about oesophageal cancer? Most people are not aware of it, don't know it.
Kijk, als het nou een algemeen bevolkingsonderzoek is, dan hoef ik dat niet te bespreken. Maar als er een screening onder een select gezelschap plaatsvindt, dan zou ik wel willen weten waarom dat ik daarbij hoor. En dan zou ik eigenlijk liever hebben dat het via mijn huisarts komt.	See, if it were general population-based screening, I wouldn't have to discuss anything. But if only a select group is screened, then I would like to know why I am part of that group. And then I would really prefer hearing it from my GP.
Transnasal endoscopy	
Ze kunnen de neus verdoven, waardoor de prikkeling in de neus weg is.	They can numb the nose, so that the tingle in the nose is gone.
Ik heb zelf wel onderzoek gehad naar mijn stembanden en dat gaat ook allemaal via je neus en best wel ver naar achter en ik vond dat eigenlijk helemaal niet zo bezwaarlijk.	I personally had a test to examine my vocal cords, which also goes through your nose and reaches quite far back. I actually didn't find that too burdensome.
Ik bedoel, dat kokhalzen wordt er niet meer of minder door lijkt me?	I mean, it won't make the gagging any better or worse, right?
Ik moet eerlijk zeggen, ja, ik bijt er wel doorheen, maar ik zie er meer tegenop als door de mond.	Honestly, well, I'll get through it with gritted teeth, but I dread it more than via the mouth.
Ja, via de mond heb je denk ik meer personeel nodig dan via de neus. Ik denk dat via de neus één op één kan.	Yes, I think you would need more staff with a test via the mouth than via the nose. I think the one via the nose can be done by one person.

Degene die er naar kijkt, die moet dat echt dus heel goed kunnen beoordelen.	The person who examines it must be able to judge it very well.
Hiermee selecteer je dus eigenlijk van tevoren, want je kunt niet een bevolkingsonderzoek doen via het ziekenhuis, dat is gewoon niet te doen. En misschien is dat met zo'n bus wel te doen. En degene bij wie niks gezien is, die komt niet in het ziekenhuis. Dus je ontlast de zorg denk ik.	This way you pre-select, because you can't perform population-based screening in a hospital, that is simply not possible. But maybe it's doable with a mobile unit. And if nothing is detected, you don't have to go to hospital. So I think it decreases the burden on health care.
Wat is de verbetering? Dat die vijf millimeter is? Het blijft door je keel. Het gaat dan eerst door je neus en in plaats van één keer moet je misschien twee keer. Het verschil zit m dan in het roesje. Ik vraag me af, is dat een wezenlijke verbetering?	What's the improvement? That it is five millimeters? It still goes down your throat. It first goes through your nose and instead of undergoing it once you may have to do it twice. The difference is in the sedation. I wonder, is that really a considerable improvement?
'Een simpel onderzoekje', daar versta ik iets anders onder.	'A simple test', I mean something else by that.
Ingestible cell-collection device	
Denk maar dat het een lekker snoepje is, en je slikt hem zo weg. Klaar. Toch?	Just imagine it's a delicious piece of candy, and it'll go right down. All done. Right?
Ik neem aan dat dat hetzelfde is als dat je nou je pil inneemt.	I assume that it is the same as taking your pills.
Ik weet het nog zo net niet. Ik denk dat het touwtje in je keel, dat is een voorwerp wat er niet hoort te zetten, dat zorgt automatisch voor heel veel irritatie en kokhalsneigingen. Ga maar na, als de tandarts achter met een spiegeltje wilt kijken en hij komt al in de buurt...	I'm not convinced. I think that the piece of string in your throat, which is something that shouldn't be there, will automatically cause a lot of irritation and gagging. It's like when the dentist uses a mirror to check your molars and he gets close…
Akelig.	Unpleasant.
Mensen gaan dat, binnen de kortst mogelijke tijd trekken ze het eruit. Dus in mijn eigen ervaring heb ik echt zoiets van: dit werkt niet.	People will pull that thing out in no time. So from my experience, I'm really like: this isn't going to work.
Maar als dat door je keel naar boven moet, dat sluit wel even de boel af.	But if that has to go up through your throat, that will close things off for a while.
Dan denk ik van: hoe kun je dan ineens 2 cm lang, hoe breed, een centimeter in omvang, en dat moet je dan maar door je keel zien te krijgen.	I'm just thinking: how can you swallow a 2 cm long, how wide is it, a centimeter in circumference? How could you get that down your throat?
Het heeft ook een kleefwerking. In hoeverre blijft je stem, hoe heten ze nou, je trilvezels van je stem daaraan kleven? Je stembanden bestaan uit hele fijne vezels. In hoeverre blijven die eraan kleven als je het sponsje terughaalt en dus beschadiging van mogelijk je stem opleveren.	It also has an adhesive effect. To what extent does your voice, what are they called, do the vibrating fibers of your voice stick to it? Your vocal cords are made up of very fine fibers. To what extent do they stick to the sponge when you retrieve it and thus possibly damage your voice?
Maar dat touwtje, ik weet niet, als ik slik, en hoe ver komt dan bij de eerste slik dat medicijn in de slokdarm? Zakt die dan uit zichzelf naar beneden, ook voorbij	But that piece of string, I don't know, if I swallow, how far does that medicine get into the oesophagus on the first swallow? Will it drop down by itself, also past my

mijn klep?	valve?
En die pil kan halverwege blijven hangen, dat je denkt: ik krijg hem niet verder.	That pill can get stuck halfway, and you'll be like: I can't get it any further.
Nou, als het met een touwtje moet en het breekt af, dan weet ik niet waar ik dan liever ben.	Well, if the piece of string snaps, I don't know where I'd rather be.
Ja, ik denk, die kanker, ja, ik heb toch het idee, dat die op een bepaald plekje begint. En dat sponsje, ja, dat schraapt cellen mee, maar hij kan de niet-gewenste cellen, kan hij dus missen. Ja, dat is dan een gemiste kans.	Yeah, I think, that cancer, Yeah, I still think that the cancer starts in a certain place. And that sponge, yeah, it scrapes cells along with it, but it may miss the cancerous cells. So that would be a missed opportunity.
Breath analysis	
Als je mocht kiezen, als ik gewoon kijk, wat zou ik het fijnste vinden? Natuurlijk het blaasonderzoek, dat is wel zo. Maar goed, die nauwkeurigheid vind ik toch wel belangrijker.	If I get to choose, let me see, what would I find the most comfortable? That would of course be the breath test, that's true. But, you know, I find accuracy more important.
Het maakt wat minder schade. Want het andere kan allemaal schade maken, een hapje verwijderen, als er net iets verkeerd gaat.	There is less risk of damage. The other tests can all cause damage, taking a biopsy, if something is just a little off.
Op zich is het een goede methode, omdat die waarschijnlijk vrij goedkoop is, daar moet je ook wel naar kijken.	In principle it is a good method, because it is probably quite affordable, you should also consider that.
En ik denk dat dit ook makkelijker wijds in te zetten is dan een slang.	And I think this is also easier to implement on a large-scale than a tube.
Want het is laagdrempelig, je kunt het dus bij de huisarts al uitvoeren.	Because it is easily accessible, you can even do it at the GP's office.
Ik word niet zo blij van zo'n test, laat ik het zo zeggen. Kijk, als de huisarts zegt: 'blaas maar even', dan doe je dat. Maar ik zal er niet gerust op zijn nadat ik heb gehoord: 'er is niks aan de hand'. Dus dat is echt wel een ding. Uiteindelijk wil je ook mensen toch wel… ja, 'zekerheid' is een groot woord, maar toch wel op een betrouwbare manier een en ander vertellen. Ik sta niet te springen hierom.	I'm not thrilled by such a test, let's put it that way. Look, if the GP says: 'just take a breath', then you'll do that. But I won't be reassured after I hear: 'there's nothing wrong'. So that's a real issue. In the end, you want to tell people, well, 'certainty' is a big word, but you still want to relay results in a reliable way. I'm not eager to participate with this test.
En als het niet betrouwbaar is, dan heb je er geen, sorry dat ik het zeg, geen reet aan.	If it isn't reliable, I'm sorry to say, but then it's completely useless.
Is dit nou voor slokdarmkanker of is dit ook al voor die… nou ben ik de naam weer kwijt, die Barrett?	Is this for oesophageal cancer or is it also for that… now I've forgotten the term again, that Barrett?
Ik was benieuwd eigenlijk: wat meet het? Het maagzuur of zo? Het zuurgehalte van de maag? Bloedspatten?	I was curious actually: what does it measure? The stomach acid or something? The acidity of the stomach? Blood spatters?
Oké dus via je longen zou je ook kunnen meten of je slokdarmkanker hebt…	Okay, so you could measure whether you have oesophageal cancer via your

Terwijl dat toch weer een ander systeem is toch?	lungs… Even though that is another system, right?
