# Supplementary material for: Dutch individuals’ views of screening for oesophageal cancer: a focus group study
Source: BMJ Open Gastroenterol. 2023 May 31;10(1):e001136. doi: 10.1136/bmjgast-2023-001136 (PMC10254588; doi:10.1136/bmjgast-2023-001136)
Supplement: Supplementary data [file bmjgast-2023-001136supp001.pdf]

Semi-structured interview guide focus group discussions

"Individuals' views of screening for oesophageal cancer"

Version 5

Views of screening for oesophageal cancer: Focus Group interview guide version 5.0 17-01-2022

PART 1. INTRODUCTION (5 min)

Welcome everyone to this group discussion. [JS introduces herself]. [LR introduces herself]. Today we are going to talk about oesophageal cancer and screening for it. It is important for us to know what the public thinks about screening for oesophageal cancer. That is why we have invited you to discuss this topic. I would like to encourage you to chat with each other today, my role will be more in the background. I will occasionally ask a question or suggest a direction, after which you can continue the conversation with each other. Everything you say is of interest to me, so there are no right or wrong answers. I can imagine that you will have questions during the conversation. I invite you to ask them to each other. We are very interested to hear your questions, but we won't be answering them during the focus group. This may sound a little frustrating, but there will be plenty of time to ask your questions at the end. This way, we don't influence the discussion.

After about an hour there will be some time to get a cup of coffee or tea. We would like to record this conversation so that we can listen to it later and transcribe it. Of course, we will keep the recordings confidential and your identity will not be revealed in reports of this group discussion. Is everyone okay with that? Do you have any questions about the group discussion before we start?

I will now start recording the conversation.

PART 2. PERCEPTIONS OF OESOPHAGEAL CANCER (10 min)

Suggested topics:

1. What comes to mind when you think of oesophageal cancer?
2. Do you think there are certain people or groups of people who are more likely to develop oesophageal cancer?
3. Which factors do you think may increase the risk of oesophageal cancer?
4. How high do you think your personal risk of oesophageal cancer is compared with people of your age?
5. Do people around you ever talk about oesophageal cancer?
6. What comes to mind when you think of heartburn?
7. What are potential consequences of heartburn?
8. Have you ever heard of Barrett's oesophagus?

Views of screening for oesophageal cancer: Focus Group interview guide version 5.0 17-01-2022

PART 3. PERCEPTIONS OF SCREENING FOR OESOPHAGEAL CANCER (50 min)

I will now give you some information about oesophageal cancer. I will use a few information graphics. Please share any questions you have with the group. They're all very informative to me.

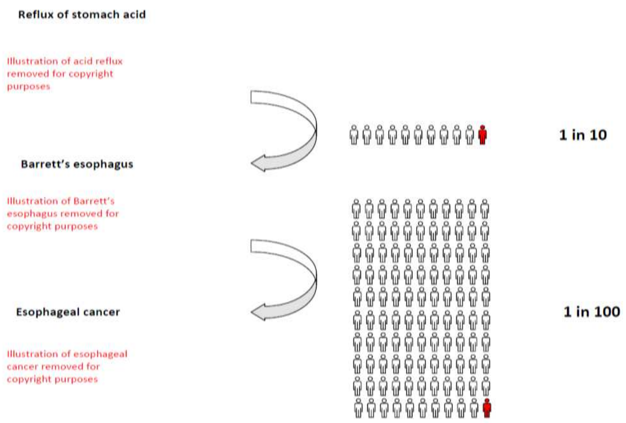

Figure 1. Information graphic 1

[Verbal information, supported by information graphic 1]

Oesophageal cancer usually develops as a result of acid reflux. When stomach acid splashes back into the oesophagus, you may experience a burning sensation in your chest and throat. The stomach acid may also irritate the oesophagus. In around 1 in 10 people, the reflux of stomach acid causes a condition called Barrett's oesophagus. People with Barrett's oesophagus have a slightly higher risk of developing oesophageal cancer than people without it. Around 1 in 100 people with Barrett's oesophagus will develop oesophageal cancer in their lifetime.

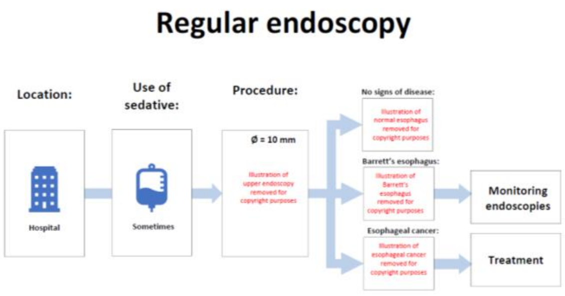

Figure 2. Information graphic 2

Sijben J, *et al. BMJ Open Gastro* 2023; 10:e001136. doi: 10.1136/bmjgast-2023-001136

[Verbal information, supported by information graphic 2]

People with severe heartburn are sometimes referred for an endoscopy by their GP. During endoscopy, a camera on a tube is entered through the mouth. The tube is about 1 centimetre wide. Sometimes a light sedative is used. The oesophagus is inspected to look for abnormalities. If a Barrett's oesophagus is suspected, oesophageal tissue samples are extracted to examine under the microscope. If Barrett's oesophagus is confirmed, the person receives regular invitations for endoscopy (surveillance) to check for signs of early cancer.

Oesophageal cancer starts on the inside lining of the oesophagus. Early stage cancer can be cut or burned away during an endoscopy. If the cancer has grown deeper into the oesophagus, the entire oesophagus is surgically removed. If late-stage cancer is detected curative treatment is no longer an option. Despite monitoring people with Barrett's oesophagus, most cases of oesophageal cancer are still detected late with limited treatment options.

An extra test might help find more people with Barrett's oesophagus and early stages of oesophageal cancer. This process is called screening. In the second hour of this discussion, I will introduce three screening tests that are currently being researched.

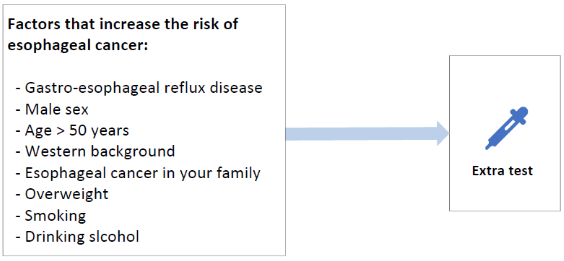

Figure 3. Information graphic 3

[Verbal information, supported by information graphic 3]

Oesophageal cancer is less common than other cancers for which we have population screening in the Netherlands. Therefore, if screening for oesophageal cancer were to be implemented, it is likely that only people with higher-than-average risk of oesophageal cancer would be invited. Factors that increase the risk of oesophageal cancer are, for example, gastro-oesophageal reflux disease, being

over 50 years old, Western background, male sex, smoking, being overweight or having a family member with oesophageal cancer.

What are your impressions of screening for oesophageal cancer after hearing this information?

Suggested topics:

1. What do you think the benefits of oesophageal cancer screening could be?
2. What do you think the drawbacks of oesophageal cancer screening could be?
3. Do you think screening for oesophageal cancer is useful? Why? Why not?
4. Please imagine you have beentold that your risk of developing oesophageal cancer is above average.
   Which thoughts come to mind?
   Which feelings does the higher risk provoke?
5. Would you like to know your personal risk of developing oesophageal cancer?
6. Please imagine you have been advised to undergo screening for oesophageal cancer.
   Which thoughts come to mind?
   Which feelings does the screening advice provoke?
7. Can you think of reasons not to participate in oesophageal cancer screening?
8. Would screening for oesophageal cancer have an impact on your life? How?
9. How would you like to be informed about the different screening options??
   Who should inform you?
10. Suppose you had to pay for the screening test yourself, how would this influence your decision to participate?
11. Would having a test for oesophageal cancer affect your daily routine (work, sport)?
12. Would you like to involve other people when making your decision about participating in oesophageal cancer screening?

We will now have a break for 5 minutes.

Views of screening for oesophageal cancer: Focus Group interview guide version 5.0 17-01-2022

PART 4. PERCEPTIONS OF SCREENING TESTS (50 min)

We will now discuss 3 screening tests for oesophageal cancer that are being researched. We are curious to see what you think of these tests. We will discuss them one by one.

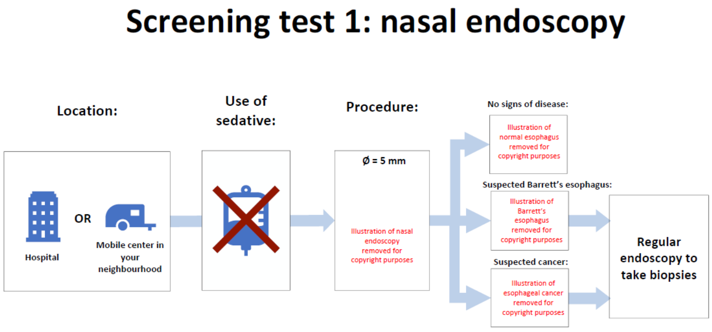

Figure 4. Information graphic 4

[Verbal information, supported by information graphic 4]

We will start with nasal endoscopy. The tube used to insert a camera in the oesophagus during this test is thin compared with regular endoscopy. This allows it to enter through the nose instead of through the mouth. The oesophagus is inspected with the camera. If there are signs of Barrett's oesophagus or oesophageal cancer, you are referred to the hospital for a regular endoscopy to take tissue samples. It would be possible to take these samples using the thin nasal tube, but these are not reliable enough.

Nasal endoscopy can be performed at a hospital or at a mobile center. The test is performed without a sedative.

1. What is your impression of nasal endoscopy?
2. Would you be interested in having a nasal endoscopy to screen for Barrett's oesophagus and oesophageal cancer? Why? Why not?

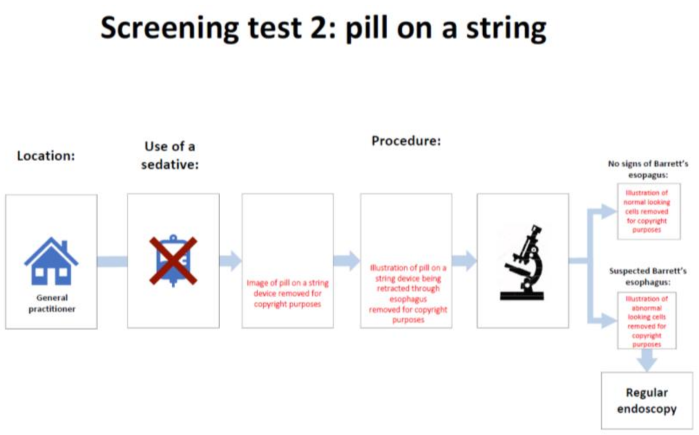

Figure 5. Information graphic 5

[Verbal information, supported by information graphic 5]

We will now discuss a test which consists of a pill on a string. The pill is about 2 cm long and can be swallowed. When the pill enters the stomach, the capsule dissolves and a sponge expands. The sponge is then pulled back through the mouth using the attached string. During withdrawal, cells of the oesophagus stick to the sponge. These cells are sent to a laboratory to check for signs of Barrett's oesophagus. If suspicious cells are seen, you are referred for a regular endoscopy to confirm the diagnosis.

The pill on a string test can be done at your GPs office. The test is performed without a sedative.

1. What is your impression of the pill on a string test?
2. Would you be interested in having the pill on a string test to screen for Barrett's oesophagus and oesophageal cancer? Why? Why not?

Screening test 3: breath test

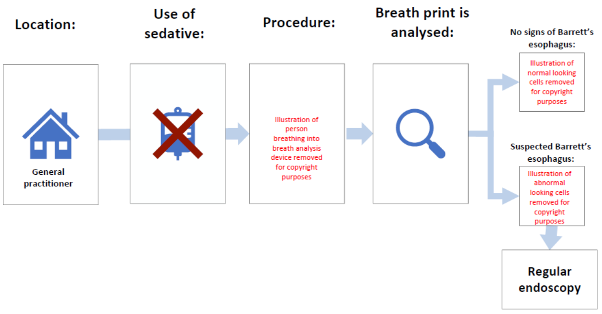

Figure 6. Information graphic 6

[Verbal information, supported by information graphic 6]

We will now discuss a breath test. A disease can change the composition of components found in exhaled breath. Analysis of these 'breath prints' may therefore be a way to diagnose Barrett's oesophagus. During the test, you breathe through an electronic device for 5 minutes. The device measures your breath print and compares it to breath prints from people with Barrett's oesophagus. If, based on this, there is a suspicion of Barrett's oesophagus, you will be referred for a regular endoscopy to confirm the diagnosis.

The breath test can be done at your GPs office. The test is performed without a sedative.

1. What is your impression of the breath test?
2. Would you be interested in having the breath test to screen for Barrett's oesophagus and oesophageal cancer? Why? Why not?

Finally, we will compare these tests to each other. The tests are not equally reliable when it comes to distinguishing people with and without Barrett's oesophagus. The regular endoscopy is the most reliable test, followed by the nasal endoscopy and the pill on a string test. More research is needed to find out how reliably the breath test can detect Barrett's oesophagus.

1. Does this information change your opinion about the tests?

The 3 new tests have in common that a suspicion of Barrett's oesophagus needs to be confirmed by a regular endoscopy.

2. How do you feel about this?
3. Which of these four tests would you prefer and why?
